# Prevalence of skin diseases among elderly prisoners in Taiwan: an examination of skin health in prison

**DOI:** 10.1186/s12877-024-05181-0

**Published:** 2024-07-06

**Authors:** Zhu Liduzi Jiesisibieke, Yu-Pei Yang, Yen-Chun Wang, Ching-Wen Chien, Tao-Hsin Tung

**Affiliations:** 1 Evidence-based Medicine Center, Taizhou Hospital of Zhejiang Province affiliated to Wenzhou Medical University, Linhai, Zhejiang China; 2 Department of Hematology, Taizhou Hospital of Zhejiang Province, Wenzhou Medical University, Linhai, Zhejiang China; 3 Department of Public Health, College of Health Science, Kaohsiung Medical University, Kaohsiung, Taiwan; 4 Institute for Hospital Management, Tsing Hua University, Shenzhen Campus, Shenzhen, China; 5 Department of Urology, Enze Hospital, Taizhou Enze Medical Center (Group), Taizhou Hospital of Zhejiang Province Affiliated to Wenzhou Medical University, Affilitated to Hangzhou Medical College, Taizhou, Zhejiang China; 6 Key Laboratory of Evidence-based Radiology of Taizhou, Linhai, Zhejiang China; 7 Taizhou Institute of Medicine, Health and New Drug Clinical Research, Taizhou, China

**Keywords:** Elderly prisoners, Prevalence, Skin diseases, Population-based study

## Abstract

**Background:**

Although prisoner health is a topic of significant importance, it has received limited attention in epidemiological studies, likely due to challenges in obtaining data. Therefore, this study aimed to investigate the prevalence of skin diseases among elderly prisoners in Taiwan.

**Methods:**

We examined the presence of skin diseases in 2215 elderly prisoners based on the International Classification of Diseases, 9th revision Clinical Modification (ICD-9-CM). Additionally, the most common types of skin diseases among elderly prisoners in Taiwan were identified.

**Results:**

The prevalence of skin diseases among prisoners was estimated to be 55.03%. Elderly men prisoners exhibited a higher prevalence of skin diseases than the women prisoners. The most common skin diseases observed were as follows: contact dermatitis and other forms of eczema; pruritus and related conditions; cellulitis and abscesses; and urticaria.

**Conclusion:**

Skin diseases were identified in more than half of the elderly prisoners. The overall quality of life of elderly prisoners can be improved by addressing their skin health, which would contribute to the fulfilment of their basic human rights.

**Clinical trials number:**

NA.

## Background

Despite the importance of prisoner health, epidemiological studies have devoted little attention to this topic. This lack of research is likely due to the challenges in obtaining data [1]. As the number of incarcerated individuals continues to increase, their health care needs also increase in magnitude [2]. According to the principle of “equivalence of care,” all prisoners have the right to appropriate health care services [3]. Although efforts have been made to achieve equality among prisoners [4, 5], considerable challenges remain [6–8]. Moreover, even after prisoners are released and reintegrated into society, they face health care discrimination from medical professionals based on their criminal records [9].

Skin diseases, particularly communicable diseases, must be properly addressed due to their potential for rapid spread among inmates, staff, and visitors [10]. Prison environments are typically subpar in term of overcrowding and hygiene issues [11, 12]. In addition to discomfort and pain, skin diseases can lead to other health complications that negatively affect mental well-being [13]. Since a significant percentage of prisoners eventually reintegrate into society, inadequate treatment of these health problems can ultimately lead to societal burdens and increased costs [14]. In prisons in Taiwan, the prevalence of skin diseases is estimated to be 42.25% [15], which is significantly higher than the 35.5% prevalence rate in the general population [16]. Ageing and declining health status make elderly prisoners particularly vulnerable to several health issues. However, the prevalence of skin diseases among elderly prisoners has rarely been studied.

This study aimed to analyse data from the Taiwan National Health Insurance Research Database (NHIRD) to investigate the prevalence of skin diseases among elderly inmates in prisons in Taiwan. By identifying the most common skin diseases, we hope to provide preventive strategies for prison healthcare staff. The objective was to enhance the health and well-being of prisoners while also addressing the need to minimize the spread of contagious skin conditions and improve overall fairness within Taiwan’s correctional facilities.

## Methods

### Data source

The Taiwan National Health Insurance (NHI) system was introduced in 1995 and is a single-payer system based on public contracts [17]. This system offers numerous advantages, such as cost-effectiveness, high efficiency, and extensive population coverage. However, it also has several shortcomings, such as short consultation times and inadequate gatekeeping for special needs [18]. To enhance the reliability of the NHIRD, certain measures have been implemented to improve the validity of disease codes and account for unmeasured confounders [19]. Since it became accessible in 2000, the NHIRD has been used in a multitude of epidemiological studies to facilitate information and knowledge sharing [20]. The NHIRD uses the International Classification of Diseases, 9th Revision Clinical Modification (ICD-9-CM). Previous studies have provided detailed information to gain a more comprehensive understanding of the NHIRD [15, 21]. Prisoners were selected for this study from this large administrative dataset, which includes data from all prisoners from January 1, 2013, to the end of the same year. The Institutional Review Board of Cheng-Hsin General Hospital approved this study (CHGH-IRB: [471]104-07), and all patient data were handled in accordance with the Declaration of Helsinki guidelines while ensuring patient anonymity.

### Study population

This study specifically focused on individuals aged ≥ 65 years. Skin diseases were assessed using the ICD-9 code range of 680–709, which was further divided into three groups to facilitate easy referencing: skin and subcutaneous tissue infections (680–686), other inflammatory conditions of the skin and subcutaneous tissue (690–698), and other diseases of the skin and subcutaneous tissue (700–709). To ensure data reliability, the prisoners were required to receive a minimum of three diagnoses within one of these groups, indicating the presence of skin diseases. Additionally, due to the lack of socioeconomic and biomarker data in the NHIRD, we can only perform a descriptive study.

### Statistical analysis

Statistical analyses were performed using SAS version 9.1 developed by SAS Institute, Inc. (Cary, NC, USA). Categorical variables and prevalence are expressed as absolute frequencies and percentages, respectively. Prevalence were calculated by dividing the number of prisoners with each subtype of skin diseases by the total number of prisoners. Sex-specific prevalence were calculated by dividing the number of prisoners with each subtype of skin diseases in men and women separately by the total number of men and women prisoners, respectively. We used medicine service times to represent specific instances where prisoners received medical treatments, including services in outpatient clinics, hospitalizations, or emergency visits. The χ^2^ test was applied to assess differences in categorical variables. Statistical significance was set at *p* < 0.05.

## Results

Among the 2215 prisoners eligible for the study, 6.4% were women, and 93.6% were men (Table 1). The average age of the women prisoners was 70.00 years, whereas the average age of the elderly men prisoners was 69.83 years (Table 1). Additionally, of the average age of elderly women prisoners with skin diseases was 69.76 years, while the average age for elderly men prisoners with skin diseases was 69.88 years (Table 1). Similar age distributions were observed between the overall elderly prison population and prisoners with skin diseases. Notably, elderly prisoners with skin diseases required medical services at a rate 1.19 times higher than that of their counterparts to address their health care requirements (Table 1).

**Table 1:** Demographic information of study population (n = 2215)

	Total			Diseases of the skin and subcutaneous tissue	
	Women (*n* = 142)	Men (*n* = 2073)		Women (*n* = 50)	Men (*n* = 1169)
Age					
Mean (standard deviation)	70.00(5.18)	69.83(5.37)		69.76(5.96)	69.88 (5.05)
Range (min-max)	65–88	65–103		65–88	65–94
Medicine Service Times (a year)					
Mean (standard deviation)	30.79(18.15)	24.80(19.06)		33.40(21.39)	29.86(20.16)
Range (min-max)	2-122	1-165		2-122	2-165

The prevalence of sex-specific skin diseases is shown in Table 2. Skin diseases affected 35.21% of the elderly women prisoners, with contact dermatitis and other eczema being the most frequently diagnosed diseases (18.31%), followed by pruritus and related conditions (7.75%), other cellulitis and abscesses (7.04%), cellulitis and abscesses of the fingers and toes (2.82%), and urticaria (2.82%). Among elderly men prisoners, the prevalence of any skin disease was 56.39%. The most common diseases were contact dermatitis and other eczema (38.06%), followed by pruritus and related conditions (13.56%), other cellulitis and abscesses (8.97%), and urticaria (7.38%). Men had a higher prevalence of skin diseases than women.

**Table 2:** Sex-specific prevalence of skin and subcutaneous tissue diseases

		Women				Men		
		n	%	mean age (S.D.)		n	%	mean age (S.D.)
**Total prisoners**		142	100.00	70.00 (5.18)		2073	100.00	69.83 (5.37)
ICD9_680–709 Diseases of the skin and subcutaneous tissue		50	35.21	69.76 (5.96)		1169	56.39	69.88 (5.05)
**ICD9_680–686 Infections of skin and subcutaneous tissue**								
ICD9_680-Carbuncle and furuncle		1	0.70	-		110	5.31	70.08 (5.13)
ICD9_681-Cellulitis and abscess of finger and toe		4	2.82	68.75 (6.85)		40	1.93	68.70 (4.32)
ICD9_682- Other cellulitis and abscess		10	7.04	70.50 (6.33)		186	8.97	70.02 (5.64)
ICD9_683-Acute lymphadenitis		0	0.00	-		1	0.05	-
ICD9_684-Impetigo		1	0.70	-		4	0.19	76.00 (11.74)
ICD9_685-Pilonidal cyst		0	0.00	-		1	0.05	-
ICD9_686-Other local infections of skin and subcutaneous tissue		1	0.70	-		88	4.25	69.68 (4.56)
**ICD9_690–698 Other inflammatory conditions of skin and subcutaneous tissue**								
ICD9_690-Erythematosquamous dermatosis		0	0.00	-		65	3.14	70.26 (5.10)
ICD9_691-Atopic dermatitis and related conditions		1	0.70	-		65	3.14	70.58 (6.16)
ICD9_692-Contact dermatitis and other eczema		26	18.31	70.31 (6.80)		789	38.06	69.95 (4.97)
ICD9_693-Dermatitis due to substances taken internally		1	0.70	-		27	1.30	70.11 (5.23)
ICD9_694-Bullous dermatoses		0	0.00	-		4	0.19	68.75 (1.26)
ICD9_695-Erythematous conditions		0	0.00	-		6	0.29	70.17 (5.38)
ICD9_696-Psoriasis and similar disorders		0	0.00	-		16	0.77	69.38 (4.21)
ICD9_697-Lichen		0	0.00	-		0	0.00	-
ICD9_698-Pruritus and related conditions		11	7.75	69.00 (3.92)		281	13.56	69.87 (5.29)
**ICD9_700–709 Other diseases of skin and subcutaneous tissue**								
ICD9_700-Corns and callosities		0	0.00	-		1	0.05	-
ICD9_701-Other hypertrophic and atrophic conditions of skin		1	0.70	-		7	0.34	70.00 (3.00)
ICD9_702-Other dermatoses		1	0.70			5	0.24	70.60 (4.51)
ICD9_703-Diseases of nail		0	0.00	-		1	0.05	-
ICD9_704-Diseases of hair and hair follicles		1	0.70	-		63	3.04	69.59 (4.83)
ICD9_705-Disorders of sweat glands		1	0.70			8	0.39	71.38 (4.72)
ICD9_706-Diseases of sebaceous glands		2	1.41	66.50 (0.71)		20	0.96	69.70 (4.97)
ICD9_707-Chronic ulcer of skin		0	0.00	-		25	1.21	70.44 (5.35)
ICD9_708-Urticaria		4	2.82	68.25 (5.19)		153	7.38	70.29 (5.18)
ICD9_709-Other disorders of skin and subcutaneous tissue		1	0.70	-		18	0.87	70.17 (4.13)

Among the participants included in this study, the prevailing skin ailments were contact dermatitis and various forms of eczema, which accounted for 36.79% of the cases. This was followed by pruritus and associated conditions (13.18%), other cellulitis and abscesses (8.85%), urticaria (7.09%), and carbuncles and furuncles (5.01%), as presented in Table 3. Men generally had a higher prevalence of skin diseases (*p* < 0.0001). In terms of prevalence, there were marked sex differences in nearly all types of skin diseases, except for cellulitis and abscesses, as well as diseases affecting hair and hair follicles.

**Table 3:** Top skin and subcutaneous diseases among elderly prisoners stratified by sex

		Total			Women			Men		*P* for X^2^ test
		*n*	%		*n*	%		*n*	%	
Total prisoners		2215	100.00		142	100.00		2073	100.00	---
ICD9_680–709 Diseases of the skin and subcutaneous tissue		1219	55.03		50	35.21		1169	56.39	< 0.0001
ICD9_680-Carbuncle and furuncle		111	5.01		1	0.70		110	5.31	0.0150
ICD9_682- Other cellulitis and abscess		196	8.85		10	7.04		186	8.97	0.4333
ICD9_686-Other local infections of skin and subcutaneous tissue		89	4.02		1	0.70		88	4.25	0.0377
ICD9_692-Contact dermatitis and other eczema		815	36.79		26	18.31		789	38.06	< 0.0001
ICD9_698-Pruritus and related conditions		292	13.18		11	7.75		281	13.56	0.0478
ICD9_704-Diseases of hair and hair follicles		64	2.89		1	0.70		63	3.04	0.0611
ICD9_708-Urticaria		157	7.09		4	2.82		153	7.38	0.0404

## Discussion

In this study, the prevalence of skin diseases among elderly prisoners was estimated to be 55.03%. The prevalence of skin diseases was higher in men than in women. Additionally, notable sex differences were observed in specific skin conditions, namely, carbuncles and furuncles, other local infections of the skin and subcutaneous tissue, contact dermatitis and other eczema, pruritus and related conditions, and urticaria.

The prevalence of skin diseases among elderly prisoners in Taiwan was notably higher than that reported in previous studies. In Taiwan, 42.25% of prisoners were found to suffer from skin diseases, with contact dermatitis and eczema being the most common (27.68%), followed by cellulitis and abscess (8.17%) [15]. In contrast, the prevalence of skin diseases in the general population is 35.5%, and the prevalence of skin diseases among the general elderly population is 28%, with dermatitis and eczema being the most common diagnoses [16]. However, making direct comparisons is difficult due to varying classification groups across studies. This underscores the importance of prioritizing the health conditions of elderly prisoners. However, it must be acknowledged that a subset of elderly prisoners, particularly those with limited literary skills, may feel fearful or embarrassed about seeking assistance [22]. Furthermore, prisoners encounter barriers that prevent them from making healthy choices, and there is a disparity between their expectations and current health policies [23, 24]. Therefore, it is crucial to implement interventions to enhance the interaction between prisoners and health care staff while ensuring safety.

Contact dermatitis and other eczema are the most prevalent skin diseases among elderly prisoners, with higher prevalence among men than among women. The typical symptoms of contact dermatitis include red rashes, itching, and irritation [25]. This phenomenon is often caused by several external factors [26]. Previous skin symptoms, smoking, advanced age, metal reactivity, sensitization to epoxy and N-acetyltransferase polymorphisms, and engagement in construction work are risk factors for contact dermatitis [27]. Approximately 80% of adults aged > 70 years have at least one type of skin disease [28]. The development of certain skin diseases is associated with a reduction in lipids and free fatty acids in the epidermis as part of the ageing process [29]. Steroids are a potential therapeutic option for contact dermatitis [30]. Consequently, we recommend ensuring the availability of medications that are necessary for treating skin diseases among older prisoners. Additionally, women are generally more inclined to seek health care services [31]. Coupled with our findings that skin diseases are more prevalent among men, it is clear that this population requires increased attention and tailored health care services for skin conditions.

The prison environment significantly influences disease prevention, particularly for communicable diseases. A study conducted in a prison in Taipei highlighted issues such as overcrowding, inadequate disaster prevention, limited medical supplies, and poor external resource connections [32]. Similarly, research in Ethiopia identified intestinal parasites as a major health concern for prisoners, linked to substandard sanitation. Improving facilities by providing tools such as adequate bathing soaps and water, disinfectants, and clean food utensils can greatly mitigate the risk of intestinal parasites [33]. In summary, maintaining a clean environment and ensuring access to basic medical and hygiene services are crucial for preventing communicable diseases, including certain skin conditions.

Our study has several strengths. First, we included data from the NHIRD in Taiwan, encompassing all elderly prisoners across the country, thus ensuring comprehensive representation. Second, although epidemiological studies typically focus on the association between risk factors and specific diseases, descriptive studies, particularly those focusing on specific groups, have been overlooked. However, descriptive research on the prevalence of skin diseases among elderly prisoners in Taiwan is limited. Our study addresses this knowledge gap and emphasizes the importance of preventing and treating skin diseases. However, this study has specific limitations. First, given that our study included only prisoners in Taiwan, the generalizability of our findings to other regions may be limited. Nevertheless, our research may inspire prison management staff to prioritize the health status of elderly prisoners. Second, based on our findings, it is evident that skin diseases are highly prevalent among elderly prisoners in Taiwan. Subsequent studies should focus on identifying the risk factors specific to skin diseases in the prison environment to develop more effective prevention strategies. Since the NHIRD does not provide information on socioeconomic status or other potential confounders, investigating these factors in future studies would be valuable. Third, ICD codes were used to categorize the diseases. However, ICD codes may not accurately represent the true disease state and can sometimes lead to misclassification bias as well. Last, it is important to note that all data in the NHIRD were anonymized, meaning that certain clinical tests, such as Tzanck testing and diascopy, were unavailable for analysis.

## Conclusion

In conclusion, this study revealed that a significant proportion of elderly prisoners reported experiencing skin disorders. Furthermore, we identified the most common skin conditions in this population: contact dermatitis and other eczema; pruritus and related conditions; and cellulitis and abscess. Notably, a higher prevalence of skin disorders was observed among men prisoners. Consequently, it may be beneficial to implement specialized intervention strategies specifically aimed at improving the skin health of men prisoners.

## Data Availability

All the data generated or analysed during this study are included in this published article.

## Abbreviations

ICD-9-CM: International Classification of Diseases, 9th revision Clinical Modification
NHIRD: National Health Insurance Research Database
NHI: National Health Insurance
